# Supercritical fluid (SCF)-assisted preparation of cyclodextrin-based poly(pseudo)rotaxanes for transdermal purposes

**DOI:** 10.1007/s13346-023-01385-w

**Published:** 2023-08-09

**Authors:** Gleidson Cardoso, Carlos A. García Gonzalez, Víctor Santos-Rosales, Stephania Fleury Taveira, Marcilio Cunha-Filho, Angel Concheiro, Carmen Alvarez-Lorenzo, Ricardo Neves Marreto

**Affiliations:** 1 Laboratory of Nanosystems and Drug Delivery Devices (NanoSYS), School of Pharmacy, Universidade Federal de Goiás (UFG), Setor Leste Universitário, Rua 240, Goiânia, GO 74605-170 Brazil; 2 Departamento de Farmacología, Farmacia Y Tecnología Farmacéutica, I+D Farma (GI-1645), Faculty of Pharmacy, Instituto de Materiales (iMATUS) and Health Research Institute of Santiago de Compostela (IDIS), Universidade de Santiago de Compostela, 15782 Santiago de Compostela, Spain; 3 Laboratory of Food, Drug and Cosmetics (LTMAC), School of Health Sciences, University of Brasilia, 70.910-900, Brasília, DF Brazil

**Keywords:** Cyclodextrin, Permeation enhancer, Solid dispersion, Microstructure

## Abstract

This study aims to investigate the effect of the preparation of solid dispersions using supercritical CO_2_ (scCO_2_) on the physicochemical properties and the performance of supramolecular gels based on polymer-cyclodextrin (CD) interactions (named poly(pseudo)rotaxanes, PPR) envisaging a transdermal administration. Solid dispersions containing Soluplus^®^, the antihypertensive drug carvedilol (CAR), and CD (αCD or HPβCD) were prepared and characterized by HPLC, XRPD, FTIR, and DSC. PPRs prepared from solid dispersions (SCF gels) and the corresponding physical mixtures (PM gels) were analyzed regarding rheology, morphology, in vitro drug diffusion, and ex vivo drug skin permeation. The application of scCO_2_ led to the loss of the crystalline lattice of CAR while preserving its chemical identity. On the contrary, αCD crystals were still present in the SCF solid dispersions. SCF gels were more uniform than their corresponding PM, and the supercritical treatment resulted in changes in the rheological behavior, reducing the viscosity. CAR in vitro diffusion was significantly higher (*p* < 0.05) for the αCD-based SCF gel than its corresponding PM gel. Drug skin permeation showed a significant increase in drug flux from CD-based SCF gels (containing αCD or HPβCD) compared to corresponding PM gels. Additionally, the pretreatment of the skin with αCD exhibited increased CAR permeation, suggesting an interaction between αCD and the skin membrane. Results evidenced that SCF processing decisively modified the properties of the supramolecular gels, particularly those prepared with αCD.

**Graphical Abstract:**

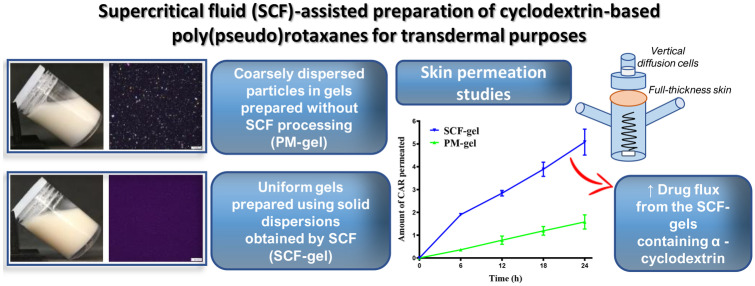

**Supplementary Information:**

The online version contains supplementary material available at 10.1007/s13346-023-01385-w.

## Introduction

Carvedilol (CAR) is a potent non-selective β-blocker with low oral bioavailability due to its poor water solubility and extensive hepatic and intestinal metabolism [1–3]. Therefore, the transdermal administration of CAR may be a promising delivery strategy to enhance treatment effectiveness [4]. However, to make this a viable possibility, it is necessary to overcome the physicochemical challenges of the molecule, which include, besides the poor aqueous solubility, a high log *P* value (greater than 3), and a relatively high molecular mass of 406.5 Da. Moreover, a successful drug transdermal administration depends on adequate skin spreadability and retention of the dosage form, as well as efficient drug permeation [5]. The drug permeation enhancement can be achieved by increasing drug thermodynamic activity in the vehicle, which could be generated by providing high loadings of the molecularly dispersed drug [6]. For this, the use of supramolecular self-assembled gels is an innovative approach for drugs such as CAR, particularly reversible polymer-cyclodextrin (CD) arrangements named poly(pseudo)rotaxanes (PPR). PPR gives rise to semisolid systems that potentially support skin retention of the formulation at the application site while increasing the drug’s apparent solubility [7, 8].

Our research group has developed CAR-loaded PPR with different polymers (Solutol^®^ or Soluplus^®^) combined with CD (α-cyclodextrin, αCD, and hydroxypropyl-β-cyclodextrin, HPβCD) [5]. These supramolecular gels were able to improve CAR solubility and in vitro release. Additionally, Soluplus^®^ PPR was obtained using CAR-HPβCD solid complexes prepared by spray drying [5] and hot-melt extrusion (HME) [6]. In both cases, an additional amorphization/complexation step in preparing supramolecular gels augmented the in vitro CAR dissolution. Marreto et al. [6] showed that the improvement in drug dissolution was related to changes in the microstructure of the supramolecular gels mainly caused by drug-CD complexation during HME. These findings demonstrated the relevance of a preprocessing step for preparing PPR gels to optimize the performance of the systems [6].

The main processes for preparing solid dispersions, i.e., the melt and the solvent evaporation methods, have limitations with undeniable practical application repercussions [9]. On the one hand, spray drying as a solvent evaporation method usually requires organic solvents, which raises safety problems and environmental damage [10]; on the other hand, HME, a melt method, uses no solvent but requires both the drug and excipient to be thermostable, miscible, and compatible at the heating temperature [11]. A promising alternative technique for producing solid dispersions and CD complexes is the supercritical fluid technology (SCF) [12]. Supercritical CO_2_ (scCO_2_) stands out as the most common choice of supercritical fluid due to its low price, non-toxicity, non-flammability, and inertness [13, 14]. Additionally, scCO_2_ has a low critical point (31.2 °C, 7.4 MPa), allowing the processing of the materials under mild conditions [12, 15]. Relevantly, scCO_2_ can also be used as a tool for product preparation and sterilization in one pot [13, 16].

The preparation of inclusion complexes with CD using scCO_2_ has been the subject of recent investigations [12]. Over 50 drugs have been complexed with CD using supercritical preparation methods, mainly the supercritical solvent impregnation method, which can be performed without organic solvents and does not require any additional drying step [12]. SCF technology was also studied as a drying method for supramolecular structures to prepare macroscopic monolithic aerogels (polyrotaxanes) [17]. The present study relies on the hypothesis that SCF processing may promote the interactions between CAR, cyclodextrins, and polymers, resulting in changes in the microstructure and properties of supramolecular gels, as well as increasing drug permeation. To the best of our knowledge, using SCF to produce PPR for transdermal drug delivery has yet to be investigated. Moreover, although CAR solid dispersions with polymer were prepared by a static scCO_2_-assisted process [10], studies on CAR complexation with CD using supercritical preparation methods have not been conducted yet.

In this work, mixtures of CAR, Soluplus^®^, αCD, or HPβCD were prepared by the scCO_2_ mixing-impregnation method, and the resulting solid dispersions were characterized using morphological, thermal, diffractometric, and spectroscopic methods. Next, the solid dispersion was used for preparing PPR supramolecular transdermal gels, and the in vitro CAR release and skin permeation were compared to those recorded from gels prepared without preprocessing. Lastly, the role of the αCD and HPβCD on skin permeation was investigated to shed light on the observed differences.

## Materials and methods

### Materials

Carvedilol (CAR, MW 406.5 Da) was from Cadila Pharmaceuticals (Dholka, India) (lot 14CVM1005). α-Cyclodextrin (αCD, Cavamax W6 Pharma^®^, lot number 601002) and hydroxypropyl-β-cyclodextrin (HPβCD, Cavitron W7 HP7^®^, lot A1411A0050, 1520 Da, molar substitution 1.03 as estimated by 1H NMR, degree of substitution 7.21) were kindly donated by Ashland Inc. (São Paulo, Brazil). Soluplus^®^ (polyvinylcaprolactam- polyvinylacetate- polyethyleneglycol, lot 844143368EO) was from BASF (São Paulo, Brazil). All the solvents and the other reagents used in this study were of analytical grade. CO_2_ (purity > 99.9%) was supplied by Nippon Gases (Madrid, Spain).

### Preparation of solid dispersions using SCF

Firstly, all formulation constituents (Table 1) were mixed in a mortar and pestle, and the mixtures were placed in a 100-mL heated stainless-steel vessel (Thar Process, Pittsburg, PA, USA). The CO_2_ was introduced to the vessel using a double-acting piston pump at 5 g/min rate until the required pressure (100 bar) at 40 °C was attained. The conditions were kept for 2 h. After that, depressurization took place at a rate of 0.5 bar/s. The resulting foamy structure matrix was then milled using a mortar and pestle. The powder fraction from 180 to 125 µm was selected for further tests. Physical mixtures (PM) of the same composition as the solid dispersions (Table 1) were prepared by placing the individual constituents into test tubes, which were sealed, and vortexed for 2 min. The Soluplus^®^/CD weight ratio was 3:1 [6].

**Table 1:** Composition of the Soluplus-based supercritical fluid dispersions (SCF) and the corresponding physical mixtures (PM). Notation: *CAR* carvedilol, *αCD* alfa-cyclodextrin, *HPβCD* hydroxypropyl-beta-cyclodextrin

**Formulation code**		**Composition (%, w/w)**			
**SCF**	**PM**	**CAR**	**αCD**	**HPβCD**	**Soluplus**^**®**^
βCD (SCF)	βCD (PM)	-	-	24.5	75.5
βCD-CAR (SCF)	βCD-CAR (PM)	10.0	-	22.0	68.0
αCD (SCF)	αCD (PM)	-	24.5	-	75.5
αCD-CAR (SCF)	αCD-CAR (PM)	10.0	22.0	-	68.0
SOL-CAR (SCF)	SOL-CAR (PM)	10.0	-	-	90.0

### Physicochemical characterization of solid dispersions

#### Differential scanning calorimetry (DSC) studies

DSC measurements were carried out using a Perkin Elmer Thermal Analyzer STA 6000 (Perkin Elmer Inc., Waltham, MA, USA) under a dynamic nitrogen atmosphere (50 mL/min) from 25 to 200 °C at a heating rate of 10 °C/min. All individual constituents, their PM, and solid dispersions were analyzed.

#### X-ray powder diffraction (XRPD)

The individual constituents, their PM, and solid dispersions were distributed on a sample holder, mounted on the vertical goniometer model PW1820/00, and analyzed in a Phillips diffractometer with cobalt radiation (CuKα *λ* = 0.15406 nm) at a voltage of 40 kV and a current of 30 mA. All XRPD profiles were measured at room temperature under a continuous scan mode (θ–2θ scan axis). The intensity data were recorded at each 0.020° in a 2θ range between 2 and 50°. The experimental setup and the following data measurements were conducted using the HighScore Plus v3.0d program.

#### Fourier-transform infrared (FTIR) spectroscopy

Fourier-transform infrared (FTIR) spectra were obtained using a Cary 630 FTIR Spectrometer (Agilent Technologies Inc., Danbury, CT, USA) with the diamond attenuated total reflectance (ATR) model with the range of 4000 to 600 cm^−1^ and the software Resolution Pro.

#### Drug content

CAR quantitation was performed by high-performance liquid chromatography with ultraviolet detection (HPLC–UV) [18]. The HPLC system was an Agilent 1260 Infinity II with a UV detector (G7114A), quaternary pump (G7111B), and auto-injector system (G7129A) (Agilent Technologies, USA). The mobile phase comprised a 50:50 (v/v) mixture of 50 mmol/L phosphate buffer (PBS) (pH 2.5 adjusted with phosphoric acid) and methanol. The flow rate was 1.0 mL/min with detection at 241 nm. The injection volume was 10 µL. Chromatographic separation was achieved at 30 °C using a ZORBAX^®^ Eclipse (Agilent Technologies, USA) XDB-C_18_ column (150 × 3.0 mm, 5 μm). Selectivity tests were conducted to evaluate the effects of the different CD and polymer on the CAR retention time and peak area.

#### Morphological characterization

The foam-like structures formed during SCF processing were evaluated by recording SEM images using a JEOL JSM 6610 (Tokyo, Japan) apparatus equipped with an energy dispersive spectrometry (EDS) X-ray detector (Thermo Scientific, Madison, USA) at LabMic/UFG. Samples were deposited on stubs and then coated with gold using a Denton Vacuum sputter coater (Desk V, Moorestown, USA) for 2 min.

### Preparation of supramolecular gels

Milled solid dispersions prepared by SCF were used to prepare supramolecular gels (SCF gels). Different amounts of each powdered solid dispersion were added to 0.05 mol/L PBS (pH 6.8) to obtain 20% (w/w) Soluplus^®^ PPR. CAR concentration was approx. 3.0% (w/w). The dispersions were kept under constant magnetic stirring at room temperature for 72 h, previously reported as sufficient for Soluplus^®^-αCD PPR formation [6].

To prepare the physical mixture gels (PM gels), firstly, a dispersion of Soluplus^®^ in 0.05 mol/L PBS (pH 6.8) was prepared under constant magnetic stirring (24 h at 300 rpm and 25 °C). Then, HPβCD (or αCD) and CAR were added, stirring the resulting mixture until complete dissolution. After adding all constituents, the mixtures were kept under constant magnetic stirring for 48 h at 300 rpm (25 °C).

### Characterization of the supramolecular gels

#### Rheological characterization

Storage (G′) and loss (G″) moduli and complex viscosity |ƞ*| of the gels were recorded in a Rheolyst AR-1000 N rheometer equipped with an AR2500 data analyzer, a Peltier plate, and cone geometry (6 cm diameter, 2.1°) (TA Instruments, Newcastle, UK). Studies were conducted at 30 °C in the angular frequency sweep (0.5 to 50 rad/s) mode. The rheometer software estimated complex viscosity, |ƞ*|, as a frequency-dependent viscosity function.

#### Morphological characterization

The supramolecular gels were observed with an Olympus BX51 optical microscope (Tokyo, Japan).

#### In vitro CAR diffusion from supramolecular gels

Drug release studies were performed using a Franz-type diffusion cell supplied by Unividros Ltd (Ribeirao Preto, São Paulo, Brazil). A dialysis membrane of regenerated cellulose with a molecular weight cutoff of 12–14 kDa was placed between the donor and the receptor chamber. An aliquot (1 g) of different formulations was added to the donor chamber. The receptor compartment was filled with 12% (w/w) Soluplus.^®^ dispersion to fulfill the sink conditions [5, 6] and kept under magnetic stirring at 300 rpm and 37 °C. Each assay was performed in triplicate for 24 h. At appropriate intervals (2, 4, 6, 8, 12, and 24 h), 0.5 mL of the receptor medium was withdrawn and immediately replaced with an equal volume of fresh dispersion. The amount released was determined by HPLC as above. The drug release kinetics was analyzed by applying zero-order (Eq. (1)) and Higuchi equations (Eq. (2))

1 $$F={F}_{0}+{k}_{0} t$$

2 $$F={k}_{H}{t}^{1/2}$$

where *F* represents the fraction of drug released over time *t*, *F*_0_ is the initial amount of drug in the gels, and *K*_0_ and *K*_H_ are the apparent rate constants for zero-order and Higuchi models, respectively.

#### In vitro CAR permeation from supramolecular gels

##### Skin obtention

Swiss mice’s full-thickness skin was used as the membrane in the permeation studies. Experimental procedures were performed according to ethical standards, especially as Normative Resolutions of the National Council for the Control of Animal Experimentation—CONCEA. The procedure was approved by the Ethics Committee on the Use of Animals/CEUA-PRPI-UFG (process number 060/21). Mice were euthanized with anesthetics, thiopental (120 mg/kg), and lidocaine (10 mg/mL), administered intraperitoneally. Next, the hairs were cut with scissors until they remained close to the skin. Fatty tissues were completely removed with a scalpel, and the full-thickness skin was separated and kept at − 25 °C for at most seven days before use.

##### Ex vivo permeation studies

Ex vivo permeation studies were carried out in Franz-type vertical diffusion cells (Unividros, Ribeirão Preto, São Paulo, Brazil). The donor compartment was filled with 1 g of the SCF or PM gels (βCD-CAR, αCD-CAR, or SOL-CAR, Table 1) corresponding to 30 mg of CAR. The receptor compartment was filled with PBS pH 3.0 and kept at 37 °C and 300 rpm. Each experiment was performed for 24 h (*n* ≥ 5). At appropriate intervals (6, 12, 18, and 24 h), 0.5 mL of the receptor medium was withdrawn and immediately replaced with an equal volume of fresh buffer. At 24 h, CAR was extracted from the full-thickness skin as described in the “CAR recovery from skin” section.

In another set of experiments, the investigation of CD contribution to CAR skin permeation was performed. Firstly, the skin was pretreated with 1 g of a CD solution (6.5%, w/w, αCD or HPβCD in PBS pH 3.0). PBS pH 3.0 was used as a control treatment. The acid pH was chosen to avoid the addition of surfactants (minimizing the undesirable effects on the biological membrane) while maximizing CAR solubility.

The CD solution was added to the donor compartment and kept in contact with the biological membrane for 6 h. Then, the solution was removed, and the donor compartment was washed with PBS pH 3.0. Finally, 1 g of the SOL-CAR SCF gel was added to the donor compartment. At appropriate intervals (6, 12, 18, and 24 h), 0.5 mL of the receptor medium was withdrawn and immediately replaced by an equal volume of PBS pH 3.0. The amount of drug permeated through the skin was determined by HPLC–UV.

##### CAR recovery from skin

After the permeation studies (24 h), drug extraction from the mouse skin was conducted. The skin was cut into small pieces and placed with 5 mL methanol in 15 mL tubes. Samples were homogenized for 2 min (Ultra-Turrax^®^ Tube Disperser, Staufen, Germany) and bath sonication for 1 h (USC 1400, Unique, Indaiatuba, Brazil). Skin homogenate was centrifuged for 10 min at 4000 rpm (SIGMA 3–18 K Centrifuge^®^, SciQuip, Shrewsbury, UK). The supernatant was filtered and analyzed by HPLC–UV. Recovery studies were performed, and drug recovery was 88.3% (± 0.7).

## Results and discussion

### Preparation and characterization of solid dispersions

In the present study, CAR-Soluplus-CD ternary solid dispersions were prepared using scCO_2_ technology, which allows the processing of the materials under mild conditions. Under such operational conditions, there was virtually no drug loss as the CAR content in the mixture was not adversely affected, and it was in the range of 96.9 to 102.9%, as determined using a validated HPLC method described in the “Drug content” section. Complete drug incorporation yields are commonly reported for formulations obtained by supercritical foaming when drugs in the formulations have moderate-to-low solubility in scCO_2_ [19, 20].

**Fig. 1:**
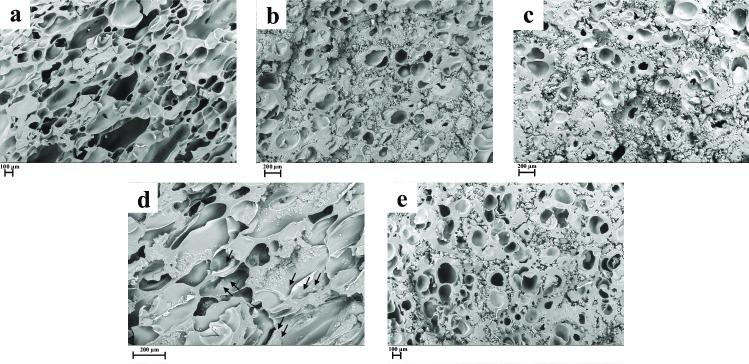
SEM images of materials processed by SCF. **a** SOL-CAR (SCF); **b** βCD (SCF); **c** βCD-CAR (SCF); **d** αCD (SCF); **e** αCD-CAR (SCF). 100 × magnification (**a**–**c** and **e**); 200 × magnification (**d**). Arrows show the αCD domains

The scCO_2_ mixing-impregnation process formed a foam-like structure (Fig. 1) due to the blowing of compressed CO_2_ associated with the volume expansion of the material during depressurization [21]. No CAR crystals were observed in the CAR-Soluplus^®^ binary mixture (SOL-CAR SCF, Table 1) (Fig. 1a), following what was reported by Djuris et al. [10], who also prepared CAR-Soluplus^®^ solid dispersions using scCO_2_. The moderate solubility of CAR in scCO_2_ and the rubbery state of Soluplus^®^ under the processing conditions favor the intimate mixing of the drug with the polymer [22, 23]. In turn, the morphology of the Soluplus^®^-HPβCD binary mixture (βCD SCF, Table 1) showed pores circumvented by an amorphous matrix (Fig. 1b) which was similar to the morphological aspect of the Soluplus^®^-HPβCD-CAR ternary system (βCD-CAR SCF, Table 1) (Fig. 1c). On the other hand, binary and ternary mixtures with αCD (αCD SCF and αCD-CAR SCF, Table 1) presented some morphological differences. Indeed, it was possible to see αCD crystals incrusted in the polymer matrix in binary mixtures (Fig. 1d). However, the ternary mixture (Fig. 1e) only showed pores circumvented by an amorphous material similar to the aspect of the ternary system containing HPβCD (Fig. 1c). It should be noted that HPβCD is an amorphous material. αCD is a crystalline compound, and both are insoluble in scCO_2_ [12]. In this study, electronic microscope micrographs did not allow clear differentiation of amorphous and crystalline structures. However, it seems that CAR and CD were well-mixed with the polymer during the SCF process.

**Fig. 2:**
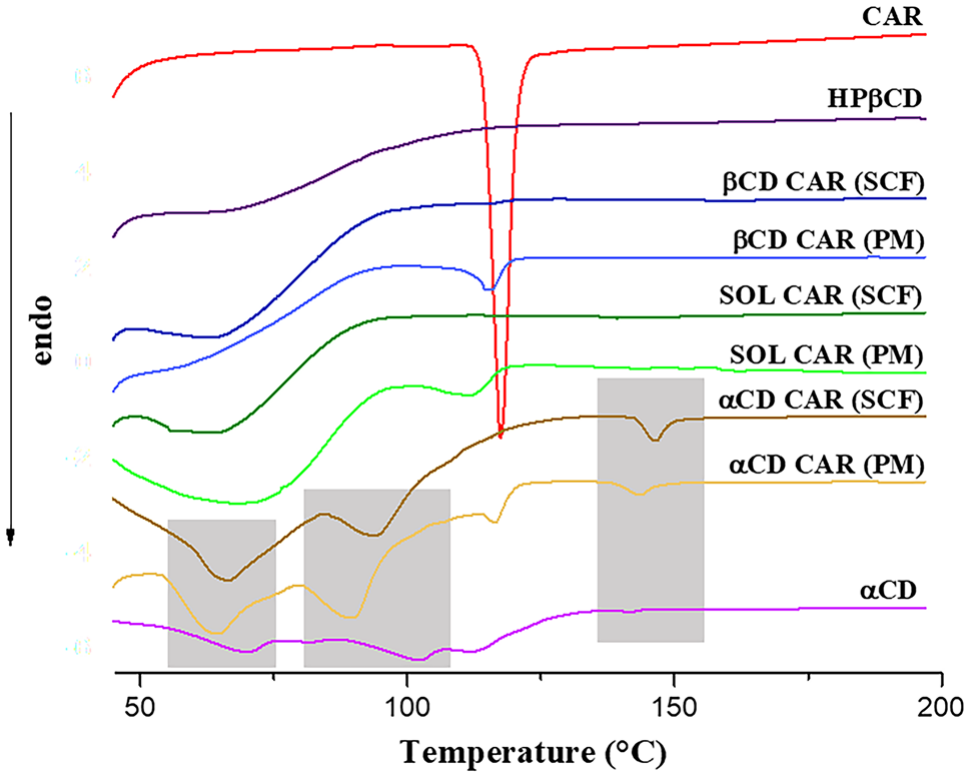
DSC curves of neat CAR, physical mixtures (PM), and SCF solid dispersions (SCF). The thermal events related to αCD are shaded

CAR and CD solid-state properties were investigated by comparing the thermal, diffractometric, and spectroscopic characteristics of SCF dispersions with their corresponding PM. DSC curves are presented in Fig. 2. The melting endotherm of neat CAR can be seen at 117.4 °C (*T*_peak_). This event was observed in PM with Soluplus^®^, HPβCD, and αCD (SOL-CAR (PM), βCD-CAR (PM), and αCD-CAR (PM)), although shifted to lower temperatures (Fig. 2), indicating interaction among the materials. The ternary solid dispersions obtained by SCF showed no drug melting event, which can be attributed to high drug-Soluplus^®^ miscibility after scCO_2_ treatment [10]. The moderate solubility of CAR in scCO_2_ may also favor the mass transport of the drug into the hydrophobic cavities of the cyclodextrin structure and its amorphization. αCD was insoluble in scCO_2_, and its mixture with CAR and Soluplus^®^ during SCF (αCD-CAR SCF) did not result in the loss of its crystalline structure suggested by the maintenance of the three dehydration endothermic events typical of neat αCD [24]. DSC curves of the Soluplus^®^ binary mixtures with CD (αCD SCF and βCD SCF) are presented in Figure S1 (supplemental material). Once more, the maintenance of the typical αCD thermal events can be noted, whereas no thermal event could be seen in neat amorphous HPβCD or its binary mixture with Soluplus^®^.

**Fig. 3:**
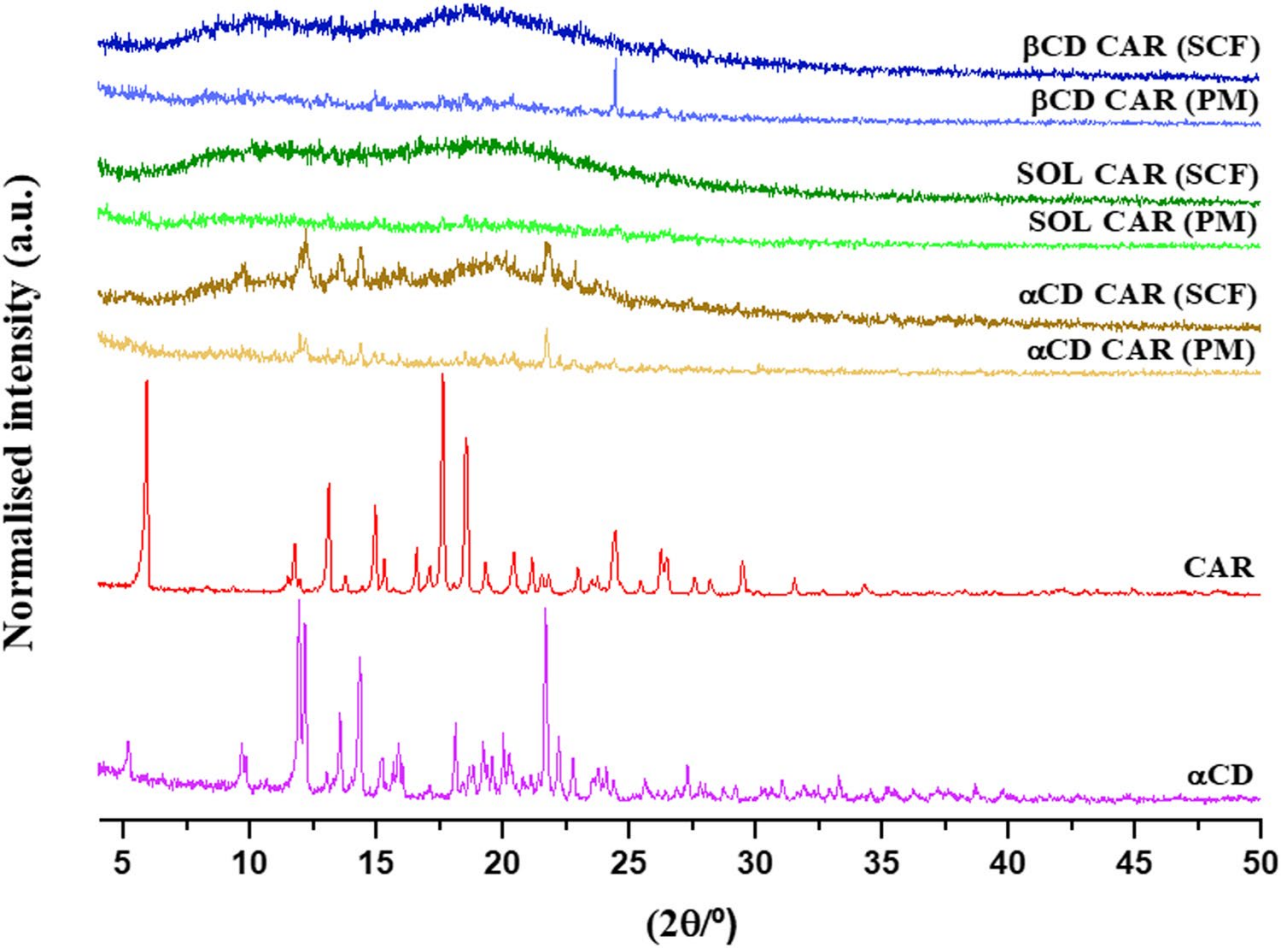
Powder X-ray diffractograms of neat CAR and αCD, SCF solid dispersions (SCF), and corresponding physical mixtures (PM)

XRPD spectra confirmed the crystalline nature of the αCD and the maintenance of its characteristic peaks in PM and SCF dispersions (αCD-CAR PM and αCD-CAR SCF) (Fig. 3). Marreto et al. [6] showed that the thermal and mechanical stress applied during the hot-melt extrusion caused the loss of αCD crystal lattice in Soluplus^®^-αCD mixtures. It seems clear that the mild processing conditions used in the SCF did not cause a similar effect. On the contrary, scCO_2_ led to complete CAR amorphization, denoted by the absence of its typical diffraction peaks in SCF samples (Fig. 3). PM samples also showed no CAR peaks, except for the mixture with HPβCD (βCD-CAR PM) that showed a Bragg diffraction at 24.34° 2ϴ. The absence of CAR characteristic peaks in most PM samples did not agree with thermal findings and could be explained by the low sensitivity of this technique for detecting CAR [18]. The binary mixtures (PM and SCF) prepared without CAR were also analyzed, and their spectra are shown in Fig. S2 (supplemental material). αCD peaks can be seen in diffractograms of SCF and PM mixtures.

**Fig. 4:**
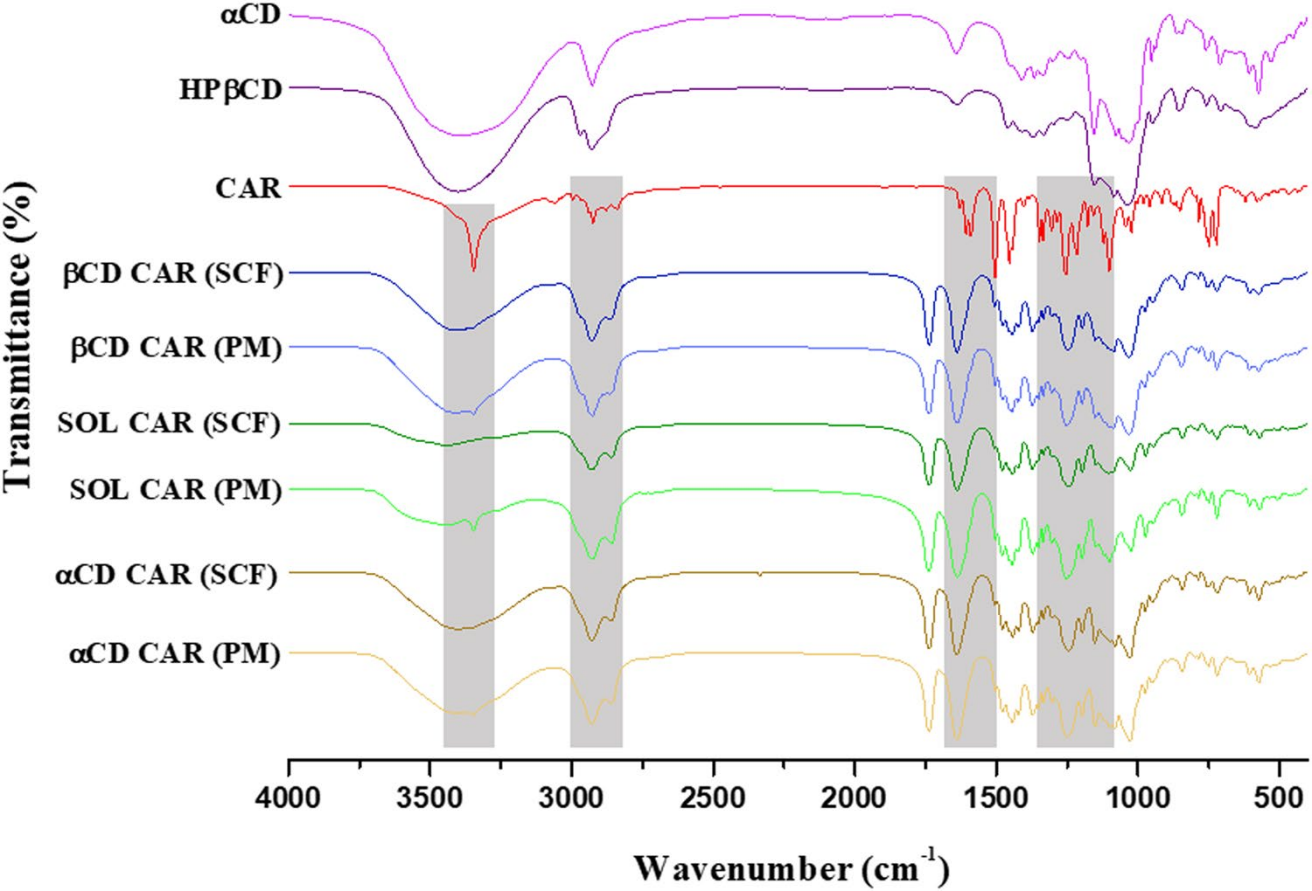
FTIR spectra of neat CAR and Soluplus^®^, SCF solid dispersions (SCF), and their corresponding physical mixtures (PM). The regions of CAR characteristic bands are shaded

The occurrence of intermolecular interactions was evaluated using FTIR spectroscopy (Fig. 4). Neat CAR had characteristic absorption bands at 3341, 2995, 2923, 1631, 1608, 1590, 1501, 1297, and 1090 cm^−1^ [25, 26]. The 3341 cm^−1^ band was ascribed to CAR combined NH and OH stretching [26]. This band was still present in the spectra of the PM, but it disappeared after SCF processing as the CAR amino group took part in drug-excipient interaction. Pesic et al. [26] also reported these changes in forming co-amorphous CAR and amino acid systems. Nevertheless, the characteristic pattern of Soluplus^®^ hampered a comprehensive observation of the CAR groups eventually involved in drug-excipient interactions. Similar behavior was seen in the spectra of the Soluplus^®^-CD binary mixtures (Fig. S3 – supplemental material).

### Preparation of SCF and PM supramolecular gels

**Fig. 5:**
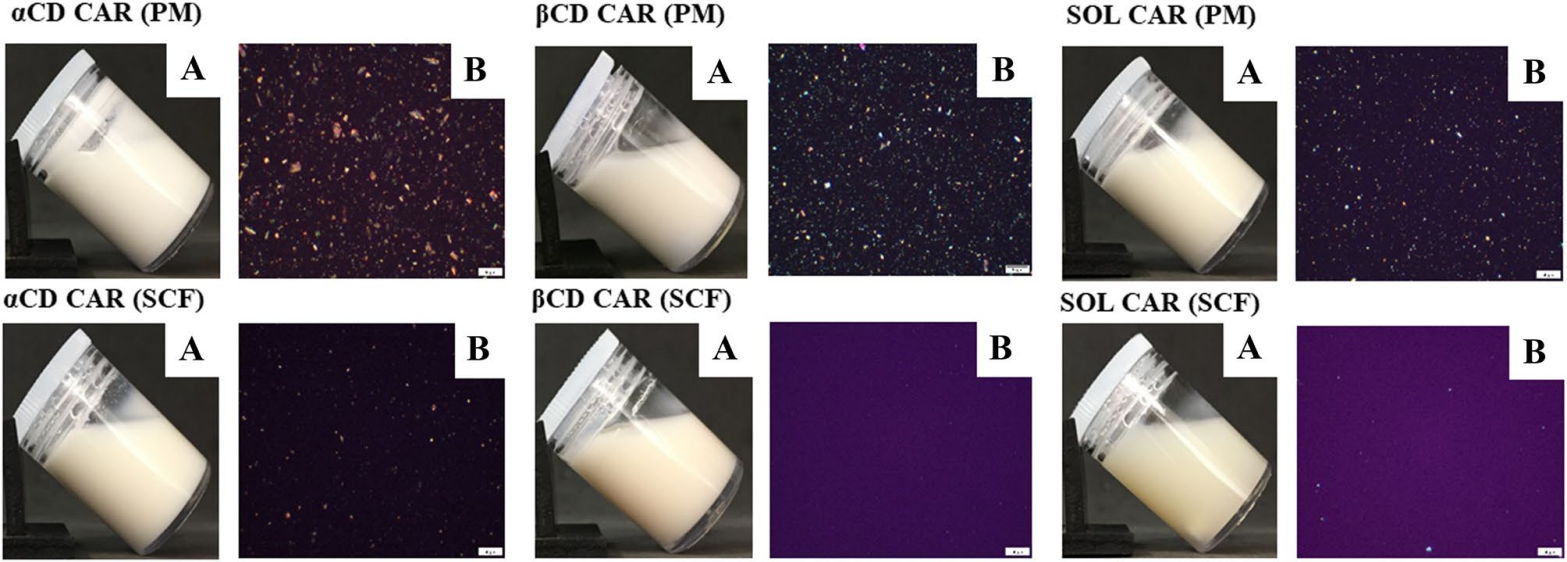
Visual appearance of CAR-loaded SCF and PM gels after magnetic stirring for 48 h. **A** Optical microscopy of gels after 48 h agitation, 40 × magnification **B**

Supramolecular gels based on Soluplus^®^-CD interactions were obtained after 48-h agitation in agreement with previous reports [5]. Optical micrographs (Fig. 5) revealed essential differences between SCF and PM gels. The latter presented coarsely dispersed particles that may be mainly ascribed to CAR crystals. This difference was already reported when supramolecular gels prepared by HME and their corresponding PM gels were compared [6]. The absence of drug crystals in gels prepared using SCF solid dispersions suggests the feasibility of this technique to improve the thermodynamic activity of the drug in the semisolid vehicle. It is important to note that a small number of particles could still be seen in optical micrographs of the αCD-CAR (SCF) and PM gels (Fig. 5b), which is related to the presence of αCD crystals, in agreement with DSC and XRPD findings.

### Rheological properties

**Fig. 6:**
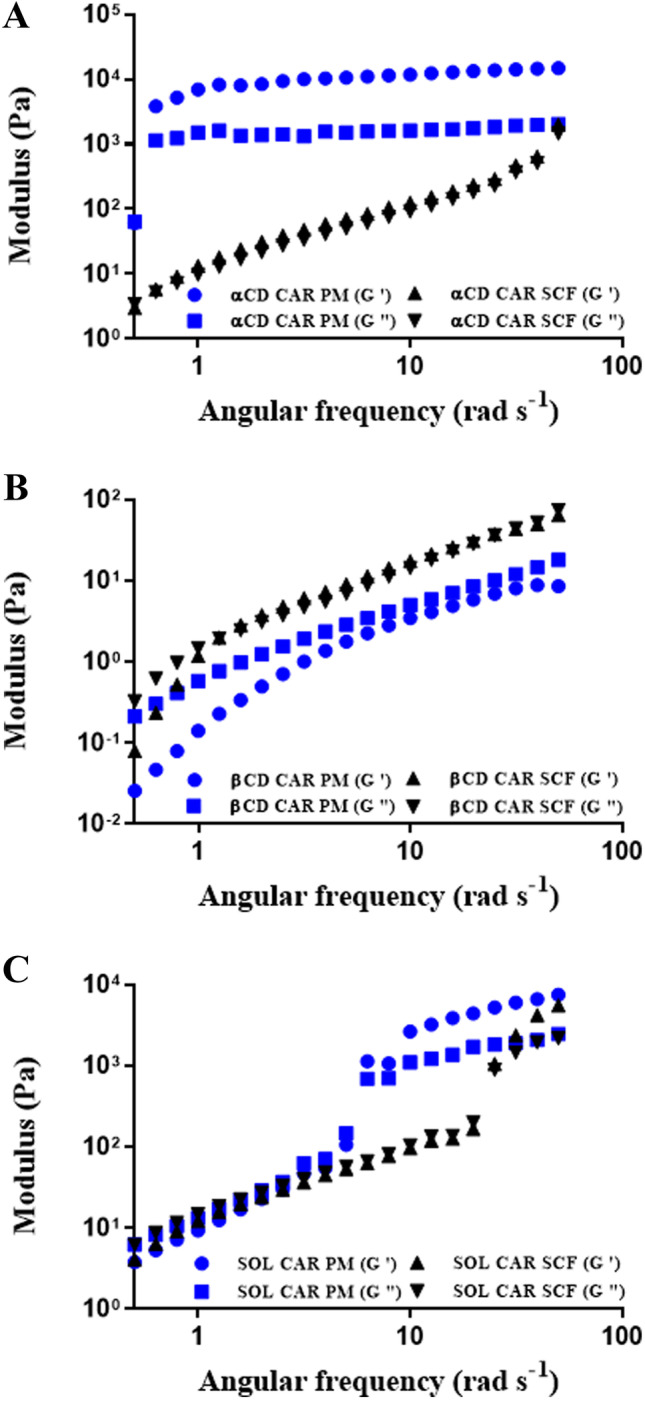
Storage modulus (G′) and loss modulus (G″) dependence on angular frequency (at 30 °C) of PM and SCF gels containing **A** CAR, Soluplus^®^, and αCD; **B** CAR, Soluplus^®^, and HPβCD; **C** CAR and Soluplus^®^

The rheological behavior of PPR supramolecular gels was described as dependent on the preparation method [6]. Hot-melt extrusion of drug-CD-polymer mixtures changed the drug dispersion state and the supramolecular assembly [6]. SCF processing also led to changes in viscosity values and viscoelastic properties of the PPR supramolecular gels (Fig. 6). αCD-CAR PM gels behaved as elastic preparations (G′ > G″). In contrast, the corresponding SCF gel showed a liquid-like behavior with superimposed moduli (G′ = G″) (Fig. 6a). Viscosity values (Fig. S4 – Supplemental material) significantly decreased after SCF processing (20 to 145-fold). PPR gels’ microstructure may have caused the observed rheological differences between PM and SCF gels prepared with αCD. Indeed, Yang et al. [27] have reported the effects of the presence of crystalline/aggregated forms of the drug clotrimazole on the rheological behavior of melted solid dispersions. These authors reported that coarsely dispersed particles are related to elastic behavior [27]. Differently, the soluble PPR composed of the Soluplus^®^-HPβCD mixture (βCD-CAR (SCF) and βCD-CAR (PM)) showed liquid-like behavior regardless of the preparation method (Fig. 6b), which suggested minor changes in the supramolecular assemblies.

Supramolecular gels prepared with only CAR and Soluplus^®^ (SOL-CAR (PM) and SOL-CAR (SCF)) showed an intermediate behavior (Fig. 6c). In this case, SCF processing caused a decrease in viscosity values. However, G′ moduli were superior to G″ at higher angular frequencies than the PM counterpart. The observed differences in gels prepared without CD can be mainly attributed to the CAR dispersion state in the preparation.

### In vitro drug diffusion

**Fig. 7:**
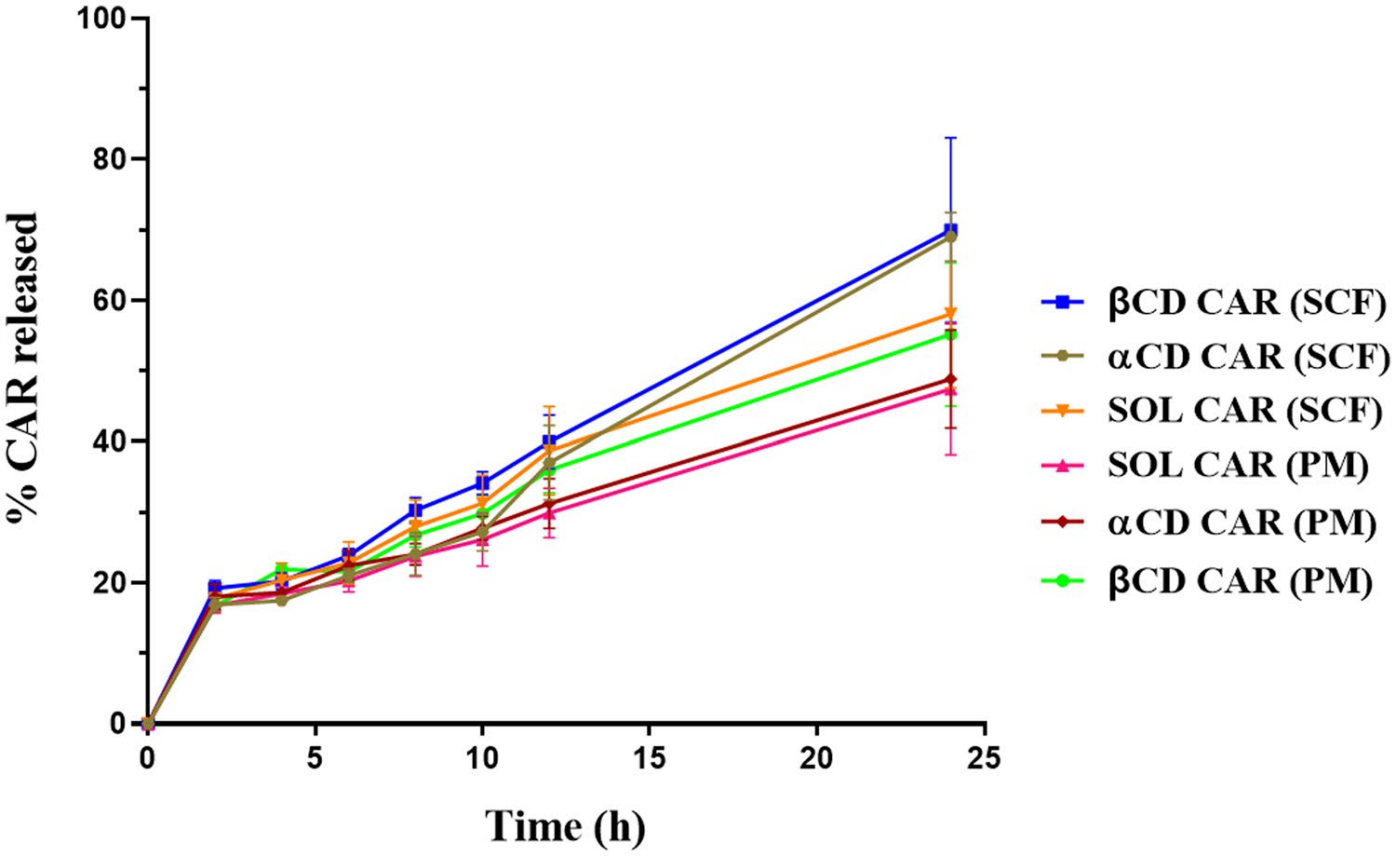
In vitro CAR release profiles from supramolecular gels prepared with SCF solid dispersions (SCF) and magnetic stirring (PM). βCD-CAR: Soluplus^®^-HPβCD-CAR ternary mixture; SOL-CAR: Soluplus^®^-CAR binary mixture; αCD-CAR: Soluplus^®^- αCD-CAR ternary mixture

In vitro CAR release profiles determined from PM and SCF gels are shown in Fig. 7, and the main release parameters are presented in Table 2. CAR release followed zero-order kinetics, and drug flux (24 h) was calculated using a zero-order equation. Drug flux was from 117.2 to 203.4 µg/cm^2^/h. No significant differences (*p* > 0.05) were observed among the PM gels. Indeed, PM gel without CD (SOL-CAR (PM)) and with αCD (αCD-CAR (PM)) presented average flux values almost identical (117.2 ± 35.6 versus 119.0 ± 28.9 µg/cm^2^/h). PM gel prepared with HPβCD (βCD-CAR (PM)) showed a higher drug flux value (144.1 ± 36.8 µg/cm^2^/h), but this increase was not statistically significant (*p* > 0.05). The presence of the CD in the formulations caused no benefits when the supramolecular gels were prepared using a simple magnetic stirring procedure without SCF processing.

**Table 2:** CAR released after 24 h (%), drug flux (*J*), and zero-order kinetics correlation coefficient (*r*) of SCF and PM gels estimated after 24-h test

**Formulation code**	**% CAR released in 24 h (SD)**^**a**^	**J (μg/cm**^**2**^**/h) 24 h (SD)**^**a**^	***r***^**b**^ **24 h**
βCD-CAR (PM)	55.2 (10.2)	144.1 (36.8)	0.9886
βCD-CAR (SCF)	70.0 (13.3)	196.9 (50.4)	0.9912
SOL-CAR (PM)	47.5 (9.3)	117.2 (35.6)	0.9945
SOL-CAR (SCF)	58.1 (10.5)	155.5 (39.2)	0.9890
αCD-CAR (PM)	48.9 (6.9)	119.0 (28.9)^c^	0.9931
αCD-CAR (SCF)	69.0 (3.4)	203.4 (13.2)^c^	0.9702

^a^SD standard deviation

^b^linear correlation coefficient of 24 h of the experiment fitted to zero-order kinetics

^c^there were significant differences between these formulations (*p* < 0.05)

All SCF gels showed an increase in average drug flux values compared to PM counterparts; however, the differences only had statistical significance when αCD was used (*p* < 0.05). Similar to what was reported for the rheological analysis, drug release data showed that SCF processing strongly affected the αCD-based gel (αCD-CAR (SCF)). In contrast, it had a lower effect on the SOL-CAR (SCF) and βCD-CAR (SCF) gels. αCD-CAR affinity is lower than βCD-CAR [28]; therefore, the loss of CAR crystalline structure in αCD-CAR (SCF) gel may have substantially favored drug inclusion in the CD cavity.

Drug flux values of αCD-CAR and βCD-CAR SCF gels were equivalent (Table 2), which was unexpected considering the differences in drug affinity for αCD and HPβCD cavities [28]. Indeed, supramolecular gels based on Soluplus^®^ mixtures with CAR and HPβCD and processed by HME have presented significantly higher drug flux values than αCD gels [6]. One possible explanation for the observed differences between both studies comprises the presence of PEG400 or PEG6000 in the HME formulations, apart from the thermal and mechanical stress during HME, which could have favored CAR inclusion in the HPβCD cavity.

Despite the lower drug flux from SCF gels than HME ones [6], SCF processing positively affected rheological and drug release properties, especially for αCD-gels. An in vitro skin permeation study was conducted to clarify the relevance of these differences.

### Ex vivo CAR skin permeation tests

Drug skin permeation is a challenge and is influenced by different skin properties (number of hair follicles, skin thickness, integrity and hydration, the distribution of fatty tissues, and others) and different formulation issues [29]. For instance, drug release, viscosity, drug saturation in the vehicle, the microstructure of the internal phase, interaction of the formulation with the skin, and many other factors could facilitate or enable drug skin permeation. Thus, in the present study, we verified whether the additional amorphization/complexation step and the changes in the microstructure of the PPR gels through SCF could enhance CAR skin permeation and retention.

**Fig. 8:**
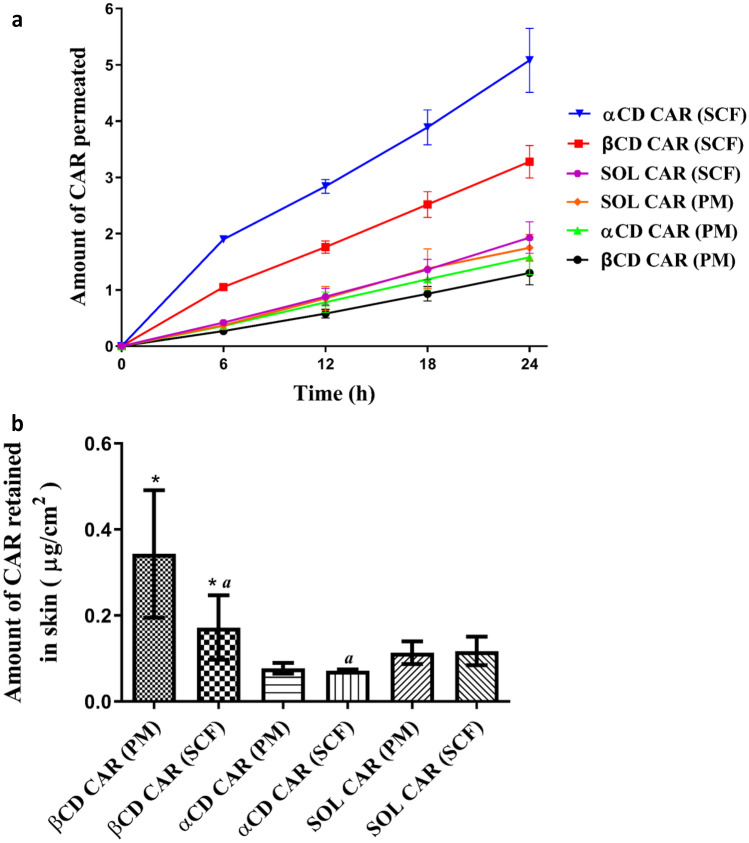
In vitro CAR permeation study. **a** CAR permeated from supramolecular gels prepared with SCF solid dispersions (SCF) and magnetic stirring (PM). **b** Amount of CAR retained in the skin after 24 h of the experiment. αCD-CAR: Soluplus^®^-αCD-CAR ternary mixture. βCD-CAR: Soluplus^®^-HPβCD-CAR ternary mixture; SOL-CAR: Soluplus^®^-CAR binary mixture

CAR skin permeation data from SCF and PM gels are summarized in Fig. 8a and Table 3. Permeation data showed a significant increase in drug flux after 24 h of the experiment from both αCD-CAR (SCF) and βCD-CAR (SCF) compared with the corresponding PM gels (*p* < 0.05). On the other hand, CAR permeation from the SOL-CAR (SCF) was similar to SOL-CAR PM one (*p* > 0.05). Despite forming an amorphous solid dispersion when the CAR-Soluplus^®^ binary mixture was processed by SCF, no apparent improvement in drug permeation was observed, suggesting the role of the drug-CD interaction after SCF treatment in the CAR permeation. In good agreement with previous reports, scCO_2_ processing ensured more efficient drug-CD inclusion complex formation than conventional and more time-consuming crushing/milling. Under scCO_2_ conditions, both the drug and the CD can be readily solubilized, favoring their interaction and ensuring that the drug is molecularly dispersed in the final solid product [30, 31]. Furthermore, from an industrial point of view, scCO2 processing might be advantageous in terms of the preservation of drug stability and scale-up of the fabrication.

**Table 3:** CAR permeated after 24 h (%), drug flux (*J*), and zero-order kinetics correlation coefficient (*r*) of SCF and PM gels estimated after 24-h test performed in full-thickness mice skin

**Formulation code**	**% CAR permeated in 24 h (SD)**^**a**^	***J*** **(μg/cm**^**2**^**/h) 24 h (SD)**^a^	***r***^**b**^ **24 h**
αCD-CAR (PM)	1.58 (0.31)	06.00 (1.31)^c^	1.0000
αCD-CAR (SCF)	4.98 (0.66)	14.47 (3.49)^c^	0.9992
βCD-CAR (PM)	1.30 (0.21)	05.11 (0.82)^d^	0.9993
βCD-CAR (SCF)	3.28 (0.29)	09.59 (1.10)^d^	0.9999
SOL-CAR (PM)	1.75 (0.24)	07.00 (1.02)	0.9982
SOL-CAR (SCF)	1.93 (0.28)	06.92 (0.96)	0.9990
**CAR permeation from SOL CAR (SCF) after 6-h treatment with αCD, HPβCD, and buffer solution (control)**			
SOL-CAR (SCF) – αCD solution	3.38 (0.89)	9.25 (1.39)^e^	0.9934
SOL-CAR (SCF) – HPβCD solution	1.91 (0.42)	5.35 (0.65)	0.9861
SOL-CAR (SCF) – control	1.72 (0.04)	5.98 (0.30)^e^	0.9945

^a^SD standard deviation

^b^linear correlation coefficient of 24 h of the experiment fitted to zero-order kinetics

^c^there were significant differences between these formulations (*p* < 0.05)

^d^there were significant differences between these formulations (*p* < 0.05)

^e^there were significant differences between these formulations (*p* < 0.05)

The effect of the different CDs on drug skin permeation is controversial. The CD can improve or reduce drug permeation depending on the experimental conditions and the vehicle type, as reviewed by Loftsson and Brewster [32]. The improvement of drug permeation may depend on the increase in diffusion through the unstirred water layer when this process is the rate-limiting step of the permeation [32]. Additionally, optimizing the CD amount in the formulation is highly relevant; otherwise, drug permeation could be reduced.

Drug flux from αCD-CAR (SCF) gel (Table 3) was higher (14.5 µg/cm^2^/h) than that calculated for βCD-CAR SCF gel (9.6 µg/cm^2^/h; *p* < 0.05). Data from the in vitro drug release study did not explain the observed differences in drug flux through mouse skin. Therefore, it could be hypothesized that the higher stability of CAR-HPβCD inclusion complexes [25] may have limited the drug availability to permeate the membrane. Previous reports have demonstrated that the formation of stable inclusion complexes with HPβCD reduced the skin permeation of methyl paraben [33]. This difference could not be seen in the drug release study because the synthetic membrane allows drug and drug-CD complex diffusion to the receptor compartment.

A penetration enhancer effect of the αCD can also explain higher drug permeation from αCD-CAR (SCF). Indeed, a significant reduction in the CAR amount retained in the skin was observed after 24 h of the experiment from αCD-CAR (SCF) gel compared with βCD-CAR (SCF) (Fig. 8b), suggesting that αCD affected drug-skin interaction. In other words, αCD interaction with skin constituents may change skin properties and, consequently, alter CAR interactions with skin, improving drug permeation and reducing drug retention.

**Fig. 9:**
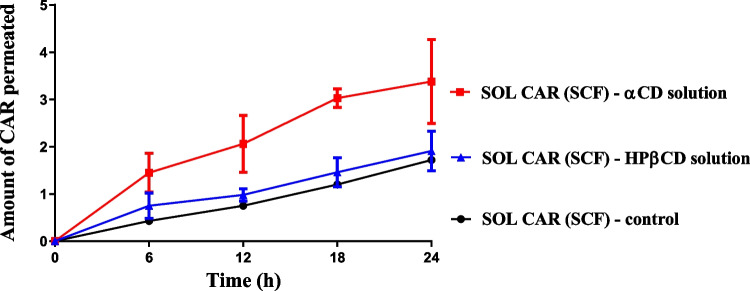
CAR permeated in mouse skin from SOL-CAR (SCF) gel after membrane pretreatment for 6 h with αCD or HPβCD solution (6.5%, w/w)

In order to better understand the role of the CDs as a potential skin permeation enhancer, a second set of permeation studies was conducted to evaluate CAR permeation from SOL-CAR (SCF) gel after pretreatment of the skin membrane with an aqueous CD solution (5%, w/w, αCD or HPβCD) (Fig. 9). A phosphate buffer was used as a control instead of a CD solution. A significant increase in CAR flux (*p* < 0.05) could only be noted when the skin was treated with αCD solution compared to the control (Table 3). This enhancer effect could not be observed when HPβCD solution was used as a pretreatment (Fig. 9, *p* > 0.05). Control pretreatment (with buffer solution) had no effect on CAR permeation from SOL-CAR (SCF) gel (*p* > 0.05).

There is scarce literature comparing the effects of αCD and βCD (or βCD derivatives) on drug skin permeation. Tenjarla et al. [34] reported an increase in miconazole permeation in human cadaver epidermis from αCD complexes compared with the drug solution. These authors also showed an increase in miconazole permeation from HPβCD complexes (versus drug solution) but using another membrane (hairless rat skin) [34]. Using two different membranes hampered a direct comparison between permeation data from drug complexes prepared with HPβCD and αCD.

Some studies have shown that αCD may form complexes with phospholipids extracted from cellular membranes [35]. However, contradictory effects of the inclusion complex formation on drug penetration through the skin have been reported. It has been conventionally stated that hydrophilic CDs do not affect drug permeation through the skin [32] and also for some drugs (e.g., ferulic acid), the inclusion complex formation has even shown to exert detrimental effects on drug permeability [36]. Differently, other studies evidenced that αCD derivatives can extract polar lipids and proteins from the skin notably promoting drug penetration [37]. Overall, the effect of αCD-drug inclusion complexes on skin penetration may be a balance between the increase in drug concentration available on the skin surface and the ratio of drug-CD and skin component-CD affinity constants. Since only free drug permeates, a highly stable inclusion complex (or an excess of CD) may explain a delayed penetration. Differently, competitive replacement of the drug from the CD cavity by skin components (lipids, proteins) may accelerate drug penetration [38]. Moreover, the extraction of certain components may weaken the barrier capability of stratum corneum. From our experiments, we can hypothesize that αCD altered the skin sufficiently to change CAR interaction with the membrane, resulting in a higher drug permeation when the αCD solution was applied to the membrane prior to the permeation experiment.

## Conclusion

The results obtained evidenced the effects of scCO_2_ processing on CAR diffusion from CD-based supramolecular gels. Even though the αCD crystalline structure was not completely lost during the supercritical process, CAR amorphization took place and the properties of the αCD-based gels were significantly improved by the SCF processing. Skin permeation studies showed the highest CAR transport through the membrane from αCD-CAR (SCF) gel, which was related, in part, to the αCD permeation enhancer effect, evidenced by the application of a skin pretreatment with αCD solution. In conclusion, SCF processing produces more uniform supramolecular gels that show increased CAR transdermal flux.

### Supplementary Information

Below is the link to the electronic supplementary material.

Supplementary file1 (DOCX 953 KB)

## Data Availability

The datasets generated during and/or analyzed during the current study are available from the corresponding author on reasonable request.
