# Supplementary material for: Supercritical fluid (SCF)-assisted preparation of cyclodextrin-based poly(pseudo)rotaxanes for transdermal purposes
Source: Drug Deliv Transl Res. 2023 Aug 9;14(1):103–15. doi: 10.1007/s13346-023-01385-w (PMC10746585; doi:10.1007/s13346-023-01385-w)
Supplement: Supplementary file 1 — Supplementary file1 (DOCX 953 KB) [file 13346_2023_1385_MOESM1_ESM.docx]

**Supercritical fluid (SCF)-assisted preparation of cyclodextrin-based poly(pseudo)rotaxanes for transdermal purposes**

Gleidson Cardoso^a^, Carlos A García Gonzalez^b^, Víctor Santos-Rosales^b^, Stephania Fleury Taveira^a^, Marcilio Cunha-Filho^c^, Angel Concheiro^b^, Carmen Alvarez-Lorenzo^b, #^, Ricardo Neves Marreto^a,^ *

^a^ Laboratory of Nanosystems and Drug Delivery Devices (NanoSYS), School of Pharmacy, Universidade Federal de Goiás (UFG), Rua 240, Setor Leste Universitário, 74605-170 Goiânia, GO, Brazil

^b^ Departamento de Farmacología, Farmacia y Tecnología Farmacéutica, I+D Farma (GI-1645), Faculty of Pharmacy, Instituto de Materiales (iMATUS) and Health Research Institute of Santiago de Compostela (IDIS), Universidade de Santiago de Compostela, Santiago de Compostela, 15782, Spain.

^c^ Laboratory of Food, Drug and Cosmetics (LTMAC), School of Health Sciences, University of Brasilia, Brasília, DF, 70.910-900, Brazil

*^*^Corresponding author:: Ricardo Neves Marreto. E-mail:* [*ricardomarreto@ufg.br*](mailto:ricardomarreto@ufg.br)*. Universidade Federal de Goiás (UFG), Rua 240, Setor Leste Universitário, 74605-170, Goiânia, GO, Brazil. Phone: +55 62 3209-6037.*

*^#^ Co-corresponding author: Carmen Alvarez-Lorenzo. E-mail:* [*carmen.alvarez.lorenzo@usc.es*](mailto:carmen.alvarez.lorenzo@usc.es)*. Universidade de Santiago de Compostela, Santiago de Compostela, 15782, Spain*.

**Fig. S1.** DSC curves of the neat Soluplus®, αCD, and HPβCD, physical mixtures (PM), and SCF solid dispersions (SCF). βCD (SCF or PM): Soluplus^®^-βCD binary mixture; αCD (SCF or PM): Soluplus^®^- αCD binary mixture.

**Fig. S2** Powder X-ray diffractograms of HPβCD, αCD and Soluplus^®^ as supplied, Soluplus^®^-CD SCF dispersions (SCF) and their corresponding physical mixtures (PM). βCD (SCF or PM): Soluplus^®^-βCD binary mixture; αCD (SCF or PM): Soluplus^®^-αCD binary mixture.

**Fig. S3**. FTIR spectra of αCD, HPβCD, and Soluplus^®^ as supplied, powdered SCF binary dispersions (SCF), and their corresponding physical mixtures (PM). βCD (SCF or PM): Soluplus^®^-βCD binary mixture; αCD (SCF or PM): Soluplus^®^- αCD binary mixture.

**Figure S4.** Viscosities of SCF and PM supramolecular gels as a function of angular frequency. αCAR: Soluplus^®^-αCD-CAR ternary mixture. βCAR: Soluplus^®^-HPβCD-CAR ternary mixture; SOL CAR: Soluplus^®^-CAR binary mixture.
